# Network analysis of associations between anthropometry, physical fitness, and sport-specific performance in young canoe sprint athletes: The role of age and sex

**DOI:** 10.3389/fspor.2022.1038350

**Published:** 2022-11-24

**Authors:** Christian Saal, Helmi Chaabene, Norman Helm, Torsten Warnke, Olaf Prieske

**Affiliations:** ^1^Faculty of Sport Science, Institute of Movement and Training Science, Leipzig University, Leipzig, Germany; ^2^Division of Training and Movement Sciences, Research Focus Cognitive Sciences, University of Potsdam, Potsdam, Germany; ^3^Olympic Testing and Training Centre Brandenburg, Potsdam, Germany; ^4^Institute for Applied Training Science, Leipzig, Germany; ^5^Division of Exercise and Movement, University of Applied Sciences for Sports and Management Potsdam, Potsdam, Germany

**Keywords:** talent identification, youth sports [MeSH], athletic performance, race time, relationship, multicollinearity

## Abstract

**Introduction:**

Anthropometric and physical fitness data can predict sport-specific performance (e.g., canoe sprint race time) in young athletes. Of note, inter-item correlations (i.e., multicollinearity) may exist between tests assessing similar physical qualities. However, multicollinearity among tests may change across age and/or sex due to age-/sex-specific non-linear development of test performances. Therefore, the present study aimed at analyzing inter-item correlations between anthropometric, physical fitness, and sport-specific performance data as a function of age and sex in young canoe sprint athletes.

**Methods:**

Anthropometric, physical fitness, and sport-specific performance data of 618 male and 297 female young canoe sprint athletes (discipline: male/female kayak, male canoe) were recorded during a national talent identification program between 1992 and 2019. For each discipline, a correlation matrix (i.e., network analysis) was calculated for age category (U13, U14, U15, U16) and sex including anthropometrics (e.g., standing body height, body mass), physical fitness (e.g., cardiorespiratory endurance, muscle power), and sport-specific performance (i.e., 250 and 2,000-m on-water canoe sprint time). Network plots were used to explore the correlation patterns by visual inspection. Further, trimmed means (μ_trimmed_) of inter-item Pearson's correlations coefficients were calculated for each discipline, age category, and sex. Effects of age and sex were analyzed using one-way ANOVAs.

**Results:**

Visual inspection revealed consistent associations among anthropometric measures across age categories, irrespective of sex. Further, associations between physical fitness and sport-specific performance were lower with increasing age, particularly in males. In this sense, statistically significant differences for μ_trimmed_ were observed in male canoeists (*p* < 0.01, ξ = 0.36) and male kayakers (*p* < 0.01, ξ = 0.38) with lower μ_trimmed_ in older compared with younger athletes (i.e., ≥U15). For female kayakers, no statistically significant effect of age on μ_trimmed_ was observed (*p* = 0.34, ξ = 0.14).

**Discussion:**

Our study revealed that inter-item correlation patterns (i.e., multicollinearity) of anthropometric, physical fitness, and sport-specific performance measures were lower in older (U15, U16) versus younger (U13, U14) male canoe sprint athletes but not in females. Thus, age and sex should be considered to identify predictors for sport-specific performance and design effective testing batteries for talent identification programs in canoe sprint athletes.

## Introduction

The regular assessment of measures of physical fitness and/or sport-specific performance in young athletes is a crucial part of talent identification and talent development in sports (1, 2). In general, talent identification (detection) and development programs aim to identify young athletes that have the potential to achieve future success in competitive sports and to select them for talent promotion programs (3). However, these programs require a multidimensional approach in which many anthropometric, physical, and physiological characteristics (e.g., body mass, muscle strength, cardiorespiratory endurance) need to be taken into account (1, 4). Thus, knowledge about the predictive value of such characteristics and their measurability is important for coaches, researchers, and athletes alike.

Important characteristics for talent identification programs in canoe sprint involve various measures of anthropometry (e.g., arm span), physical fitness (e.g., cardiorespiratory fitness), and sport-specific skills (e.g., pull-push action) (5, 6). Earlier studies used linear regression models in an attempt to explain sport-specific race times by anthropometric and fitness measures (7–10). For instance, Fry and Morton (7) examined the role of a set of anthropometric (e.g., body mass, sitting height) and physical fitness parameters (e.g., cardiorespiratory fitness, muscle strength, muscular endurance) in predicting the race time across 500 m−42 km in competitive kayakers. The regression model could explain a high proportion of race time variance for 500 m (r^2^ = 0.83), 1,000 m (r^2^ = 0.92) and 10,000 m (r^2^ = 0.90). In a recent study, Gäbler et al. (10) aimed at determining the combination of demographic (e.g., sex, discipline), anthropometric (e.g., standing body height, skeletal muscle mass), and physical fitness variables (e.g., muscular endurance, muscle power) that most accurately predict canoe sprint performance (i.e., 500- and 2,000-m race time) in young canoe sprint athletes. The authors reported that the combination of demographic (i.e., discipline), anthropometric (i.e., skeletal muscle mass), and physical fitness data (i.e., muscle power and/or muscular endurance) in the linear regression model provides the largest explained variance for 500 m (i.e., r^2^ = 0.88) and 2.000 m (r^2^ = 0.69) race time. Overall, depending on the race distance, discipline, or performance level, studies revealed trivial-to-high associations between anthropometric, physical fitness, and sport-specific performance measures (0 ≤ r ≤ 0.7). Additionally, anthropometric and/or physical fitness measures explained medium-to-high levels of variance of sport-specific performance in canoe sprint athletes (40% ≤ r^2^ ≤ 92%) (8, 10, 11).

In addition to the identification of predictive characteristics (i.e., demographics, anthropometrics) and/or performance variables, it is always worth examining the inter-item correlation of such multivariate datasets. Analyzing the inter-item correlations is useful when it comes to building or improving a statistical model and/or reducing the complexity of the data structure for reasons of multicollinearity. For instance, in their regression analysis, Gäbler et al. (10) had to remove measures such as body mass, standing body height, or maximal (handgrip) strength to reduce bias due to multicollinearity. Of note, it can be speculated that multicollinearity, and therefore the shared variances, among tests, is not constant across age and/or sex as patterns of system growth (e.g., neural, hormonal, general) and trainability (i.e., potential to adapt to training) can be characterized as specific and non-linear. For instance, the development of body height follows a double-sigmoid growth pattern in girls and boys, with rapid gains in infancy, slower yearly gains through childhood, and a second rapid gain at puberty (12). In terms of physical fitness, muscle strength (i.e., handgrip strength) and muscle power (i.e., countermovement jump height) were larger by 13 and 6%, respectively, in 13-year-old compared with 12-year-old male athletes from different sports (13). Notably, the same fitness parameters increased by 23 and 19%, respectively, from 14-year-old to 15-year-old male athletes (13). Interestingly, while young female athletes showed similar fitness differences between 12- and 13-year-olds (i.e., 16 and 6%, respectively), differences were significantly lower between 14- and 15-year-olds (i.e., only 2 and 3%, respectively) when compared with males (13). In line with these findings, differences in weightlifting performance from 12/13 to 14/15-year-old athletes amounted to 53% in males but only 14% in females (14). These results indicated that maturation and/or trainability of muscle strength and power is sex-specific. In contrast, linear speed performance did not significantly differ between young male vs. female runners of different age categories (U8–U16 years) (15).

Moreover, in addition to the empirical findings, the youth physical development model introduced by Lloyd and Oliver (16) suggested that fitness components are not equally important throughout the stages of long-term athlete development. While muscle strength should be a priority at all stages for males and females, attention to endurance and metabolic conditioning is given as the child approaches adulthood, and at no stage, it is seen as the main focus of an individual's training (16). Consequently, measures of anthropometry, physical fitness, and sport-specific performance and the respective inter-item correlation may specifically depend on age and/or sex potentially affecting the accuracy and structure of statistical models. In support of this, Haga et al. (17) examined the relationship between measures of physical fitness (i.e., endurance, muscle strength, linear speed) and general motor performance, as assessed by the “*Movement Assessment Battery for Children”* [a set of generic motor tests to identify impairments in motor performance of children (18)], in children and adolescents aged between 4 and 16 years. The results showed that the correlation coefficients between physical fitness and motor performance were significantly lower with increasing ages (4–6 years: r = 0.56; 11–12 years: r = 0.44; 15–16 years: r = 0.20). It was argued that the execution of physical fitness tasks may be more demanding at a younger age. Therefore, the fitness tasks appear to be more dependent on general motor performance (“motor competence”) (17).

Thus, the present study aimed to analyze the inter-item correlations between anthropometric, physical fitness, and sport-specific performance data as a function of age and sex in young canoe sprint athletes. Based on existing studies (12, 13, 17), we hypothesized that inter-item correlation between anthropometric, physical fitness, and sport-specific performance data may depend on the factors age and sex.

## Materials and methods

### Experimental procedure and participants

We used a cross-sectional observational study design to analyze anthropometric, physical fitness, and sport-specific performance data obtained during a national talent identification program for young German canoe sprint athletes. Standardized measurements were annually scheduled in the preparation period (i.e., end of September/beginning of October) of the athletes at an Olympic Testing and Training Center. All male and female athletes aged 12–16 years (i.e., age categories: student [under (U)13, U14], cadet [U15, U16]) who participated in the talent identification program in the federal state of Brandenburg between 1992 and 2019 were included for the analysis. Sixty-one percent of the athletes attended more than one measurement during the observation period (i.e., 2 measurements: 29%, 3 measurements: 18%, 4 measurements: 14%). Participants included 618 males (kayak: *n* = 355; canoe: *n* = 263) and 297 females (kayak only). The study complied with the Declaration of Helsinki and approval was granted by the Brandenburg Canoe Federation to analyze and publish the preexisting collected data.

### Assessment of anthropometry

Standardized testing protocols were applied for the assessment of standing body height, body mass, and arm span of young athletes. Additionally, sitting body height (i.e., height from the bottom of the seat to the top of the head) was specifically determined for young kayakers, whereas kneeling body height (i.e., height from the bottom of the ground to the top of the head during kneeling position) was measured for the young canoeists.

### Assessment of physical fitness

#### Muscle power

To determine muscle power, participants completed an underhand ball throw test. High test-retest reliability was reported previously with an intraclass correlation coefficient (ICC) of 0.95 (19). In an upright standing position, athletes hold a steel/rubber ball with both hands in front of the body. The mass of the ball was 3 kg for the girls and 5 kg for the boys. From the standing position, the athletes had to perform an underhand countermovement swing which was immediately followed by throwing the ball as far as possible. After the push-off, the feet had to remain on the ground. Three trials were performed, and the best trial in terms of maximal horizontal distance was used for analysis.

#### Muscular endurance

For muscular endurance, mechanical work was recorded during 2-min bench press and bench pull exercises. Test-retest reliability was high for both bench press (ICC = 0.94) and bench pull (ICC = 0.96) tests (10). For each exercise, participants had to perform as many repetitions as possible with a loaded barbell within 2 min. Athletes and their coaches selected the load of the barbell based on previous performance in an attempt to maximize mechanical work. A wire connected the barbell to a displacement transducer to register the total distance covered. The displacement transducer operated on the principle of a digital optical encoder. Finally, the mechanical work for both exercises was summarized and used for further analysis.

#### Cardiorespiratory endurance

As a measure of cardiorespiratory endurance, the on-land running time over 800 m for female athletes and 1,500 m for male athletes was determined on an outdoor track (400-m) with a stopwatch. High test-retest reliability was reported for this protocol (10). The athletes in the respective age groups started from a standing position after an acoustic signal was given by the tester. One trial was allowed and used for analysis.

#### Linear speed

For the assessment of linear speed, the on-land sprinting time over 30 m on an indoor track was recorded using photoelectric sensors (Tag Heuer, model CP505, La Chaux-de-Fonds, Switzerland). High test-retest reliability was reported (ICC = 0.94) (10). The start was performed individually with a run-up of 5 m (i.e., flying). The best out of three trials in terms of the fastest running time was included in the analysis.

All fitness scores were transformed using an interval-scaled scoring system to provide identical measurement units.

### Assessment of sport-specific performance

Sport-specific performance was quantified using on-water canoe sprints over a distance of 250 and 2,000 m. Race time in the respective discipline boat (i.e., kayak, canoe) was recorded with a stopwatch. The participants had to complete the 250 m trial first (i.e., pairwise start) followed by a 30-min break and the 2,000 m trial. The 2.000 m time trial was completed alone on a 1,000 m track with a “u-turn” to account for possible effects of weather conditions (e.g., wind). The individual test values of the sport-specific performance were transformed using an interval-scaled scoring system to provide identical measurement units.

### Statistical analyses

An inter-item correlation matrix was calculated for each age category (i.e., U13, U14, U15, U16) and sex (i.e., males, females) including the anthropometric, physical fitness, and sport-specific performance data. Network plots were used to explore the correlation patterns by visual inspection. Subsequently, absolute Pearson's correlation coefficients of single associations were used in a one-way ANOVA design on trimmed means (μ_trimmed_) to check for global differences between mean correlation coefficients of age category and sex in each discipline (i.e., kayak, canoe). Pairwise comparisons were conducted on each discipline (i.e., male/female kayak, male canoe) using Yuens trimmed means test. Effect sizes of ξ = 0.10, 0.30, and 0.50 correspond to small, medium, and large effects (20). An alpha level of 0.05 was used to define statistical significance. All analyses were conducted in R version 4.2.0 with the packages (20–23).

## Result

**Figure 1:**
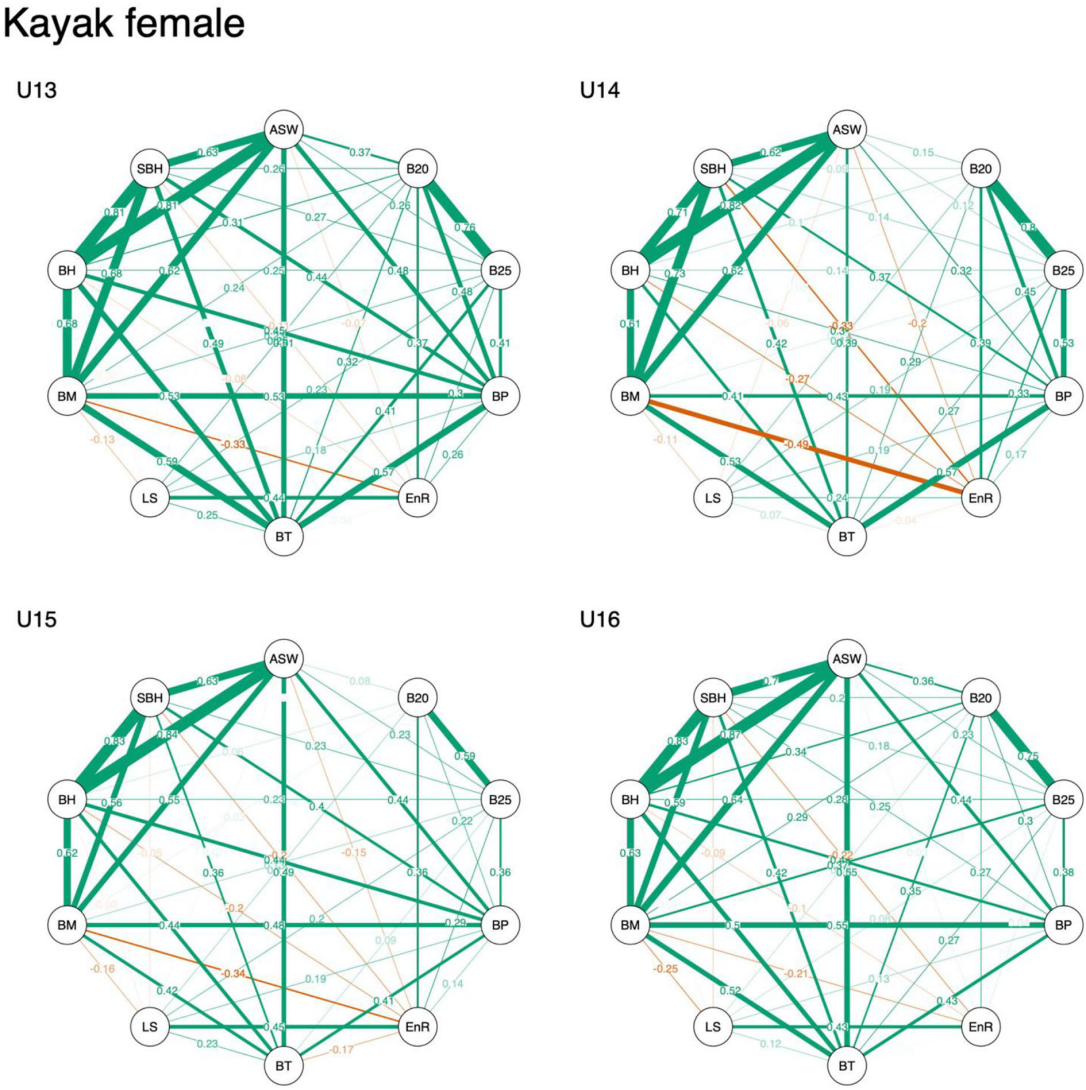
Network representation of a correlation matrix of different test items of anthropometric, physical fitness, and sport-specific performance measures in young female kayakers. ASW, arm span width; B20, 2,000 m on-water race; B25, 250 m on-water race; BP, bench press and bench pull; EnR, 800 m run; BT, underhand ball throw test; LS, 30 m linear sprint; BM, body mass; BH, body height; SBH, sitting body height.

**Figure 2:**
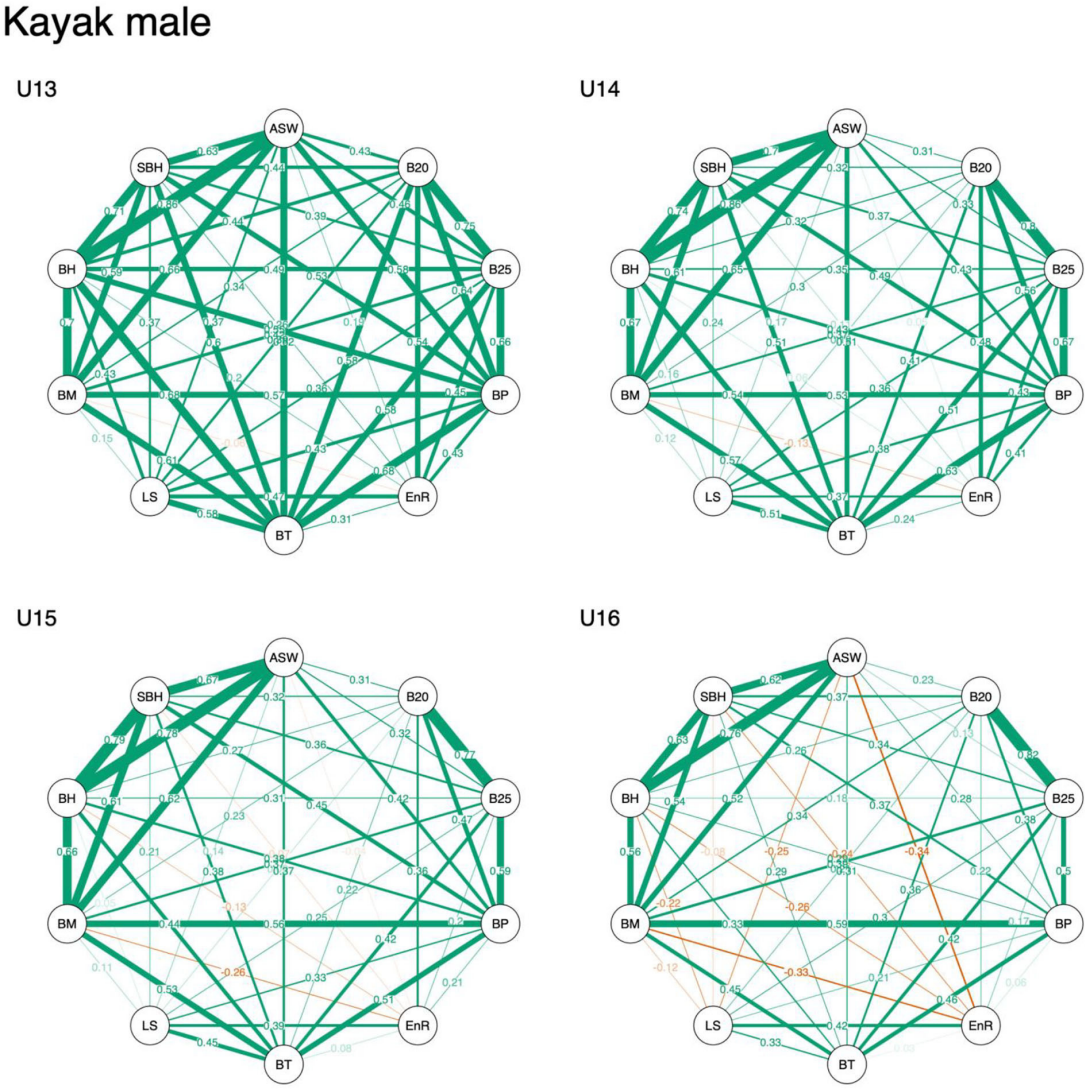
Network representation of a correlation matrix of different test items of anthropometric, physical fitness, and sport-specific performance measures in young male kayakers. ASW, arm span width; B20, 2,000 m on-water race; B25, 250 m on-water race; BP, bench press and bench pull; EnR, 1,500 m run; BT, underhand ball throw test; LS, 30 m linear sprint; BM, body mass; BH, body height; SBH, sitting body height.

**Figure 3:**
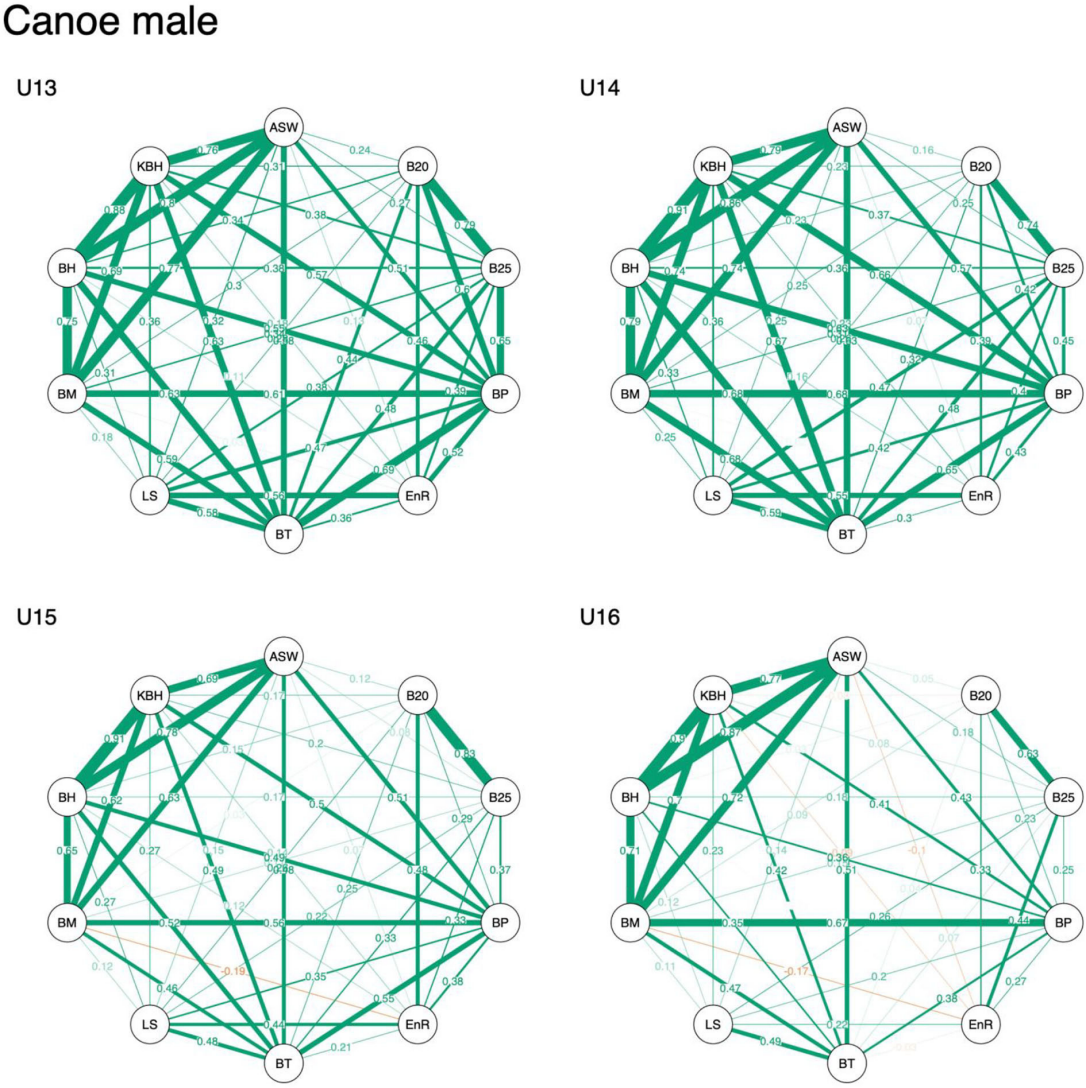
Network representation of a correlation matrix of different test items of anthropometric, physical fitness, and sport-specific performance measures in young male canoeists. ASW, arm span width; B20, 2,000 m on-water race; B25, 250 m on-water race; BP, bench press and bench pull; EnR, 1,500m run; BT, underhand ball throw test; LS, 30 m linear sprint; BM, body mass; BH, body height; KBH, kneeling body height.

Descriptive data are displayed in Tables 1–3. Overall, results indicated that correlation coefficients between anthropometric, physical fitness, and sport-specific performance ranged from −0.34 to 0.86 in male kayakers, −0.19 to 0.91 in male canoeists, and −0.49 to 0.87 in female kayakers. Visual inspection revealed that associations of anthropometric measures (i.e., standing body height, body mass, arm span, and sitting body height) remained stable across age categories, irrespective of sex. In contrast, associations of physical fitness and sport-specific performance measures decreased with increasing age, particularly in males (Figures 1–3).

**Table 1:** Anthropometric, physical fitness and sport-specific performance measures in young female kayakers.

**Outcomes**	**U13**	**U14**	**U15**	**U16**
*Anthropometry*
Standing body height [cm]	164.7 ± 6.4	168.0 ± 6.0	169.0 ± 6.1	170.3 ± 6
Body mass [kg]	55.6 ± 7.9	59.7 ± 8.3	60.7 ± 7.3	63.5 ± 6.7
Sitting body height [cm]	84.6 ± 3.5	86.4 ± 3.6	87.5 ± 3.5	88.6 ± 3.3
Arm span width [cm]	164.4 ± 7.3	168.2 ± 7.2	169.5 ± 7.3	170.0 ± 7.3
*Physical fitness*
Underhand ball throw test [pts]	53.9 ± 11.4	60.2 ± 10.8	62.4 ± 9.8	64.2 ± 11.6
Bench press/pull [pts]	74.9 ± 18.1	89.5 ± 18.1	96.1 ± 19.1	107.0 ± 18.1
800-m run [pts]	65.9 ± 20.4	70.0 ± 19.8	71.8 ± 20.6	73.5 ± 21.3
30-m sprint [pts]	51.6 ± 5.1	53.5 ± 7.3	54.1 ± 4.1	54.4 ± 4.3
*Sport-specific performance*
250-m time trial [pts]	72.0 ± 12.9	79.6 ± 10.4	84.9 ± 9.4	88.3 ± 8.1
2,000-m time trial [pts]	85.9 ± 14.6	95.5 ± 12.4	102.1 ± 13.9	105.8 ± 9.7

Data represent mean ± standard deviation.

**Table 2:** Anthropometric, physical fitness and sport-specific performances measures in young male kayakers.

**Outcomes**	**U13**	**U14**	**U15**	**U16**
*Anthropometry*
Standing body height [cm]	167.6 ± 7.6	174.5 ± 6.8	178.8 ± 6.4	180.3 ± 6.9
Body mass [kg]	56.3 ± 8.5	63.6 ± 8.4	69.7 ± 8.9	73.2 ± 7.3
Sitting body height [cm]	83.7 ± 5.0	87.7 ± 4.9	91.1 ± 3.7	92.5 ± 3.3
Arm span width [cm]	168.5 ± 8.3	176.2 ± 7.7	180.7 ± 6.6	183.6 ± 6.7
*Physical fitness*
Underhand ball throw test [pts]	60.8 ± 14.5	77.0 ± 15.4	88.9 ± 13.7	96.0 ± 14.7
Bench press/pull [pts]	90.8 ± 24.5	122.7 ± 28.6	146.1 ± 34.2	166.0 ± 32.2
800-m run [pts]	77.0 ± 24.5	93.9 ± 23.6	102.1 ± 23.4	113.3 ± 18.1
30-m sprint [pts]	53.8 ± 7.2	59.3 ± 6.5	62.7 ± 4.2	64.8 ± 3.9
*Sport-specific performance*
250-m time trial [pts]	81.3 ± 12.7	93.3 ± 11.5	100.1 ± 9.4	105.3 ± 8.4
2,000-m time trial [pts]	96.1 ± 16.8	109.1 ± 16.5	117.7 ± 14.5	122.4 ± 12.5

Data represent mean ± standard deviation.

**Table 3:** Anthropometric, physical fitness and sport-specific performances measures in young male canoeists.

**Outcomes**	**U13**	**U14**	**U15**	**U16**
*Anthropometry*
Standing body height [cm]	164.5 ± 7.7	171.2 ± 7.6	176.8 ± 6.2	179.8 ± 6.2
Body mass [kg]	52.4 ± 8.5	59.8 ± 8.9	67.3 ± 9.1	70.2 ± 8.8
Kneeling body height [cm]	122.0 ± 5.9	127.3 ± 6.2	131.8 ± 4.5	134.2 ± 4.2
Arm span width [cm]	164.8 ± 9.0	172.6 ± 9.2	179.4 ± 8.2	182.6 ± 7.5
*Physical fitness*
Underhand ball throw test [pts]	54.5 ± 13.3	68.8 ± 14.1	82.6 ± 12.9	90.1 ± 18.1
Bench press/pull [pts]	75.5 ± 19.5	100.9 ± 25.6	128.9 ± 30.4	150.4 ± 33.1
800-m run [pts]	73.7 ± 23.6	91.9 ± 21.9	103.5 ± 24.3	112.8 ± 20.0
30-m sprint [pts]	52.7 ± 5.8	57.4 ± 5.7	61.6 ± 4.2	63.4 ± 4.5
*Sport-specific performance*
250-m time trial [pts]	62.5 ± 19.6	79.3 ± 15.3	88.8 ± 12.8	96.3 ± 11.9
2,000-m time trial [pts]	74.9 ± 24.0	96.5 ± 18.7	110.0 ± 16.2	117.8 ± 14.6

Data represent mean ± standard deviation.

**Figure 4:**
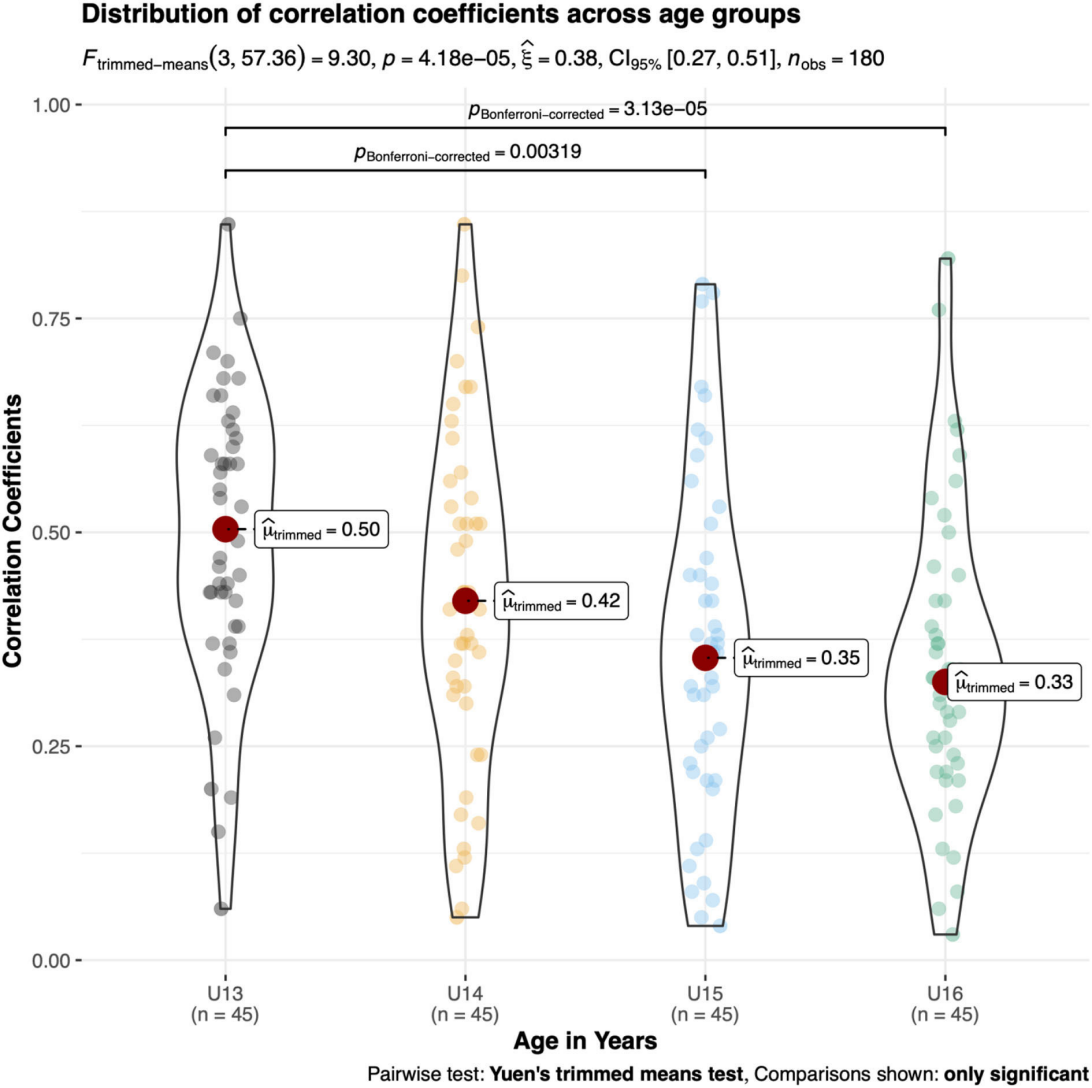
*Post-hoc* tests on trimmed means of different age categories of absolute inter-item correlation coefficients in young male kayakers.

**Figure 5:**
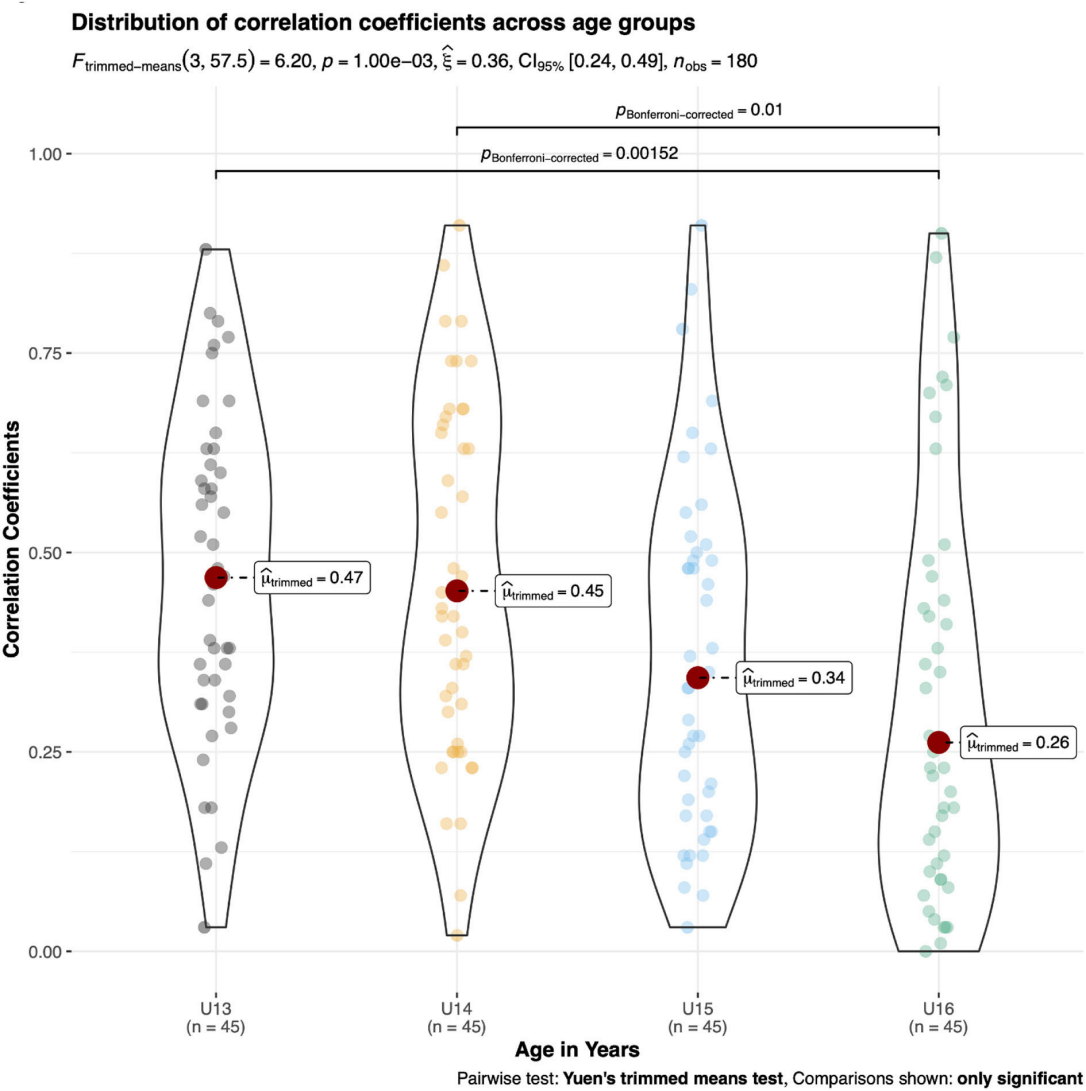
*Post-hoc* tests on trimmed means of different age categories of absolute inter-item correlation coefficients in young male canoeists.

**Figure 6:**
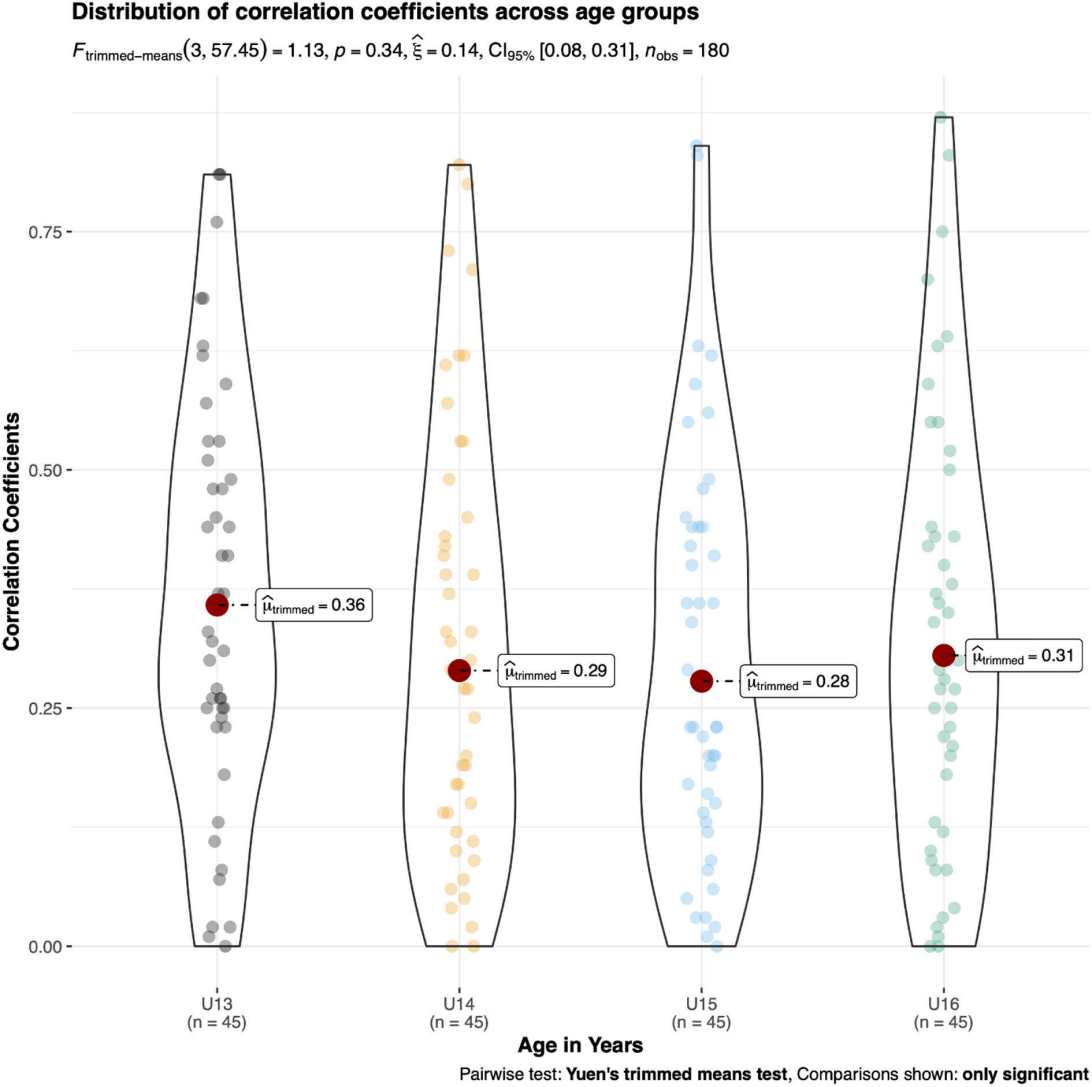
*Post-hoc* tests on trimmed means of different age categories of absolute inter-item correlation coefficients in young female kayakers.

The ANOVA outcomes indicated statistically significant differences for μ_trimmed_ across age groups in male kayakers [F_(3, 57.36)_ = 9.30, *p* < 0.01, ξ = 0.38, 95%CI (0.27, 0.51)] and male canoeists [F_(3, 57.5)_ = 6.20, *p* < 0.01, ξ = 0.36, 95%CI (0.24, 0.49)]. In male kayakers, U13 displayed a significantly larger correlation coefficient (μ_trimmed_ = 0.50) compared with U15 and U16 (μ_trimmed_ ≤ 0.35) (Figure 4). In male canoeists, U16 displayed a significantly smaller correlation coefficient (μ_trimmed_ = 0.26) compared with U13 and U14 (μ_trimmed_ ≥ 0.45) (Figure 5). No statistically significant differences in μ_trimmed_ between age groups were found for female kayakers [F_(3, 57.45)_ = 1.13, p = 0.34, ξ = 0.14, 95%CI (0.08, 0.31)] (Figure 6). When comparing the differences of μ_trimmed_ between male and female kayakers, a significant effect was observed [F_(2, 212.32)_ = 7.31, *p* < 0.01, ξ = 0.21, 95%CI (0.13, 0.32)] with significantly larger correlation coefficients in males than in females.

## Discussion

To the best of our knowledge, this is the first study that examined inter-item correlations of anthropometric, physical fitness, and sport-specific performance measures considering (chronological) age and sex in young canoe sprint athletes. We hypothesized that inter-item correlation between anthropometric, physical fitness, and sport-specific performance data are dependent on the factors age and sex. Our main findings indicated that (i) the trimmed means of inter-item correlation coefficients (μ_trimmed_) were significantly lower with increasing age (i.e., ≥ U15) in young male canoeists/kayakers, (ii) there were no statistically significant differences in μ_trimmed_ between age groups in young female kayakers, and (iii) μ_trimmed_ were significantly larger in young male compared with female kayakers.

### Role of age on inter-item correlations in male kayakers/canoeists

Previous studies in canoe sprint athletes demonstrated trivial-to-large associations between anthropometric, physical fitness, and sport-specific performance measures (8, 10, 11, 24). In line with the literature, small-to-large measures of μ_trimmed_ in young male kayakers/canoeists were reported in the current study. Moreover, supporting our hypothesis, the main findings showed that the association between anthropometric, physical fitness, and sport-specific performance measures in young canoe sprint athletes is modulated by the factors, age and sex. More specifically, mean correlation coefficients were significantly lower in male cadets (i.e., U15/U16) versus student (i.e., U13/U14) canoe sprint athletes with small-to-medium correlations in cadets vs. medium-to-large correlations in students. Functionally, these results indicate that the transfer from one anthropometric, physical fitness, and sport-specific performance measure to another was significantly lower in male cadets vs. student canoe sprint athletes. In other words, the anthropometric, physical fitness, and sport-specific tests tend to be more independent of each other in young male canoe sprint athletes with increasing age. This is partly in line with findings in children and adolescents aged 4–16 years where correlation coefficients between physical fitness and general motor performance, a surrogate measure of motor competence, decreased with increasing ages (17). Authors suggested that the technical demand to execute physical fitness tasks such as running (i.e., reduced Cooper test) or jumping (i.e., standing broad jump) may be lower in older children/adolescents due to the increasing proficiency in performance. Similar results were reported in a systematic review and meta-analysis examining the effects of age on the associations between variables of balance and lower-extremity muscle strength/power in healthy individuals (25). Specifically, the authors revealed small associations between balance and measures of muscle strength/power, irrespective of age. However, correlation coefficients between measures of steady-state balance (i.e., maintaining a steady position while walking) and maximal strength were significantly larger in children compared with young adults (25). It has been shown that motor task automation is lower in children (i.e., early in practice or low levels of movement experience) with a relatively unspecific control of motor tasks when compared with young adults (26). Thus, transfer effects between different motor tasks may be lower in young adults compared with children, when the control of motor tasks becomes more specific (e.g., due to training) (25). In fact, according to long-term training programs, there is a shift in the ratio of multilateral (i.e., unspecific) to sport-specific training content with increasing age in young athletes (27). More precisely, while multilateral-dominant training programs are recommended in the early stages of athlete development (60% multilateral and 40% specific), the proportion of sport-specific training contents should be higher in later stages (up to 20% multilateral and 80% specific) (27). Further, studies also indicated that associations between anthropometric variables and physical fitness/sport-specific performances are modulated by (chronological/biological) age (28, 29). For instance, correlation coefficients between anthropometric measures such as standing or sitting body height and 30 m sprint characteristics (step length/frequency, ground contact time) were significant and medium-to-large in (prepubertal) boys aged 12 years, but non-significant and trivial in (postpubertal) boys aged 15 years (29). Moreover, the body mass index and the medicine ball throw test were significant predictor variables for 7m throwing velocity in a multiple linear regression model in male U17 handball players (28). In U19 and U21 athletes, however, the only predictors of 7 m throwing velocity were 30 m and 10 m sprint performances, respectively (28). It was argued that the effect of maturation was more important for physical fitness/sport-specific performance measures at younger ages (29). Therefore, anthropometric, physical fitness, and sport-specific performance data appear to be more independent in male cadet vs. student canoe sprint athletes in our study due to growth- and/or training-related higher levels in motor performance proficiency in the older athletes. Additionally, the higher maturational status in cadets may have also contributed to the lower mean correlation coefficients (i.e., multicollinearity) in male cadets vs. student canoe sprint athletes. From a practical standpoint, it can be speculated that more specific testing protocols should be integrated into talent identification programs for young male canoe sprint athletes as they get older.

### Role of age on inter-item correlations in female kayakers

In contrast to the findings in young male canoe sprint athletes (kayakers and canoeists), multicollinearity was smaller and consistent across ages in young female kayakers. More specifically, no statistically significant differences in mean inter-item correlation coefficients between the different age categories in female athletes were observed. It can be speculated that sex-specific effects on growth/maturation (12, 30) and/or trainability of different fitness measures (13, 14, 31) may have contributed to the different findings in males and females noted in this study. For instance, females usually experience adolescent growth earlier than boys and progress at a faster rate compared with boys (12, 30). Further, gains in maximal strength (i.e., weightlifting performance) from 12–13-year-old to 14–15-year-old athletes amounted to 53% in male but only 14% in female athletes (14). In terms of cardiorespiratory endurance (i.e., 800 m running), sex-specific differences evolved from 4.8% at the age of 11 years to 15.7% at the age of 18 years in favor of males (31). Taking these findings into account, the young female kayakers in our study may have reached higher levels of maturation and/or training-related motor performance proficiency already at earlier chronological ages (i.e., student-athletes) compared with their male counterparts. Consequently, mean correlation coefficients (i.e., multicollinearity) were consistently small-to-medium across all age categories in young female canoe sprint athletes.

## Limitations

We have to acknowledge a few limitations of this study. First, we used a cross-sectional study design. This design is the best way to determine prevalence and allows for assessing multiple outcomes (32). Although cross-sectional studies are used to infer causation, they do not allow for cause-and-effect relations to be accurately identified (32). Further, we did not control for training volume that could have affected the development of physical fitness and sport-specific performance in young athletes. In fact, Opstoel et al. (33) showed that young athletes aged 9–11 years with better physical fitness and motor performance spend more hours per week in their sport compared with children with lower fitness and motor performance (33). Furthermore, according to the regulations of the national canoeing federation, chronological age but not maturity status was used as a variable to classify athletes. Of note, maturation is a non-linear process, which is why there is often a discrepancy between chronological age and maturation among young athletes (12, 30). This is a major challenge for talent identification/development programs in youth sports where competitions are mainly regulated by chronological age groups (13). Lastly, assessors were not the same throughout the entire observation period of 27 years, which could have affected the variability of the data. However, standard tests adopted by the national federation for talent identification were used over the years and the assessors were coaches experienced with the measurements, reducing the risk of systematic bias.

## Conclusion

This study showed that the inter-item correlation pattern (i.e., multicollinearity) of anthropometric, physical fitness, and sport-specific performance measures is influenced by age and sex in young canoe sprint athletes. We found lower multicollinearity in male cadets vs. student-athletes. Growth- and/or training-related higher levels in motor performance proficiency in older athletes may be responsible for making anthropometric, physical fitness, and sport-specific performance data more independent in male cadets vs. student-athletes. In contrast, young female kayakers may have reached higher levels of maturation and/or training-related motor performance proficiency already at earlier ages (i.e., student-athletes), potentially explaining small-to-medium inter-item correlation coefficients across all age categories. Researchers and practitioners need to consider the effects of age and sex on inter-item correlations when it comes to identifying predictors for sport-specific performance and designing testing batteries for talent identification programs in canoe sprint athletes.

## Data availability statement

The data analyzed in this study is subject to the following licenses/restrictions: The datasets generated and/or analyzed during the current study are not publicly available. Upon request, the corresponding author will share the dataset. Requests to access these datasets should be directed to CS, Christian.saal@uni-leipzig.de.

## Ethics statement

Ethical approval was not provided for this study on human participants because we retrospectively analyzed preexisting, anonymous data of a national talent identification program for young German canoe sprint athletes. The study complied with the Declaration of Helsinki and approval was granted by the Brandenburg Canoe Federation to analyze and publish the preexisting collected data. Written informed consent to participate in this study was provided by the participants' legal guardian/next of kin.

## Author contributions

CS, NH, and OP substantially contributed to conception and design of the study. OP organized the database. CS performed the statistical analysis. CS and OP wrote the first draft of the manuscript. All authors contributed substantially to the interpretation of the data, contributed to manuscript revision, read, and approved the submitted version.

## Funding

We acknowledge support from Leipzig University for Open Access Publishing.

## Conflict of interest

The authors declare that the research was conducted in the absence of any commercial or financial relationships that could be construed as a potential conflict of interest.

## Publisher's note

All claims expressed in this article are solely those of the authors and do not necessarily represent those of their affiliated organizations, or those of the publisher, the editors and the reviewers. Any product that may be evaluated in this article, or claim that may be made by its manufacturer, is not guaranteed or endorsed by the publisher.
